# Supplementary material for: Complex‐centric proteome profiling by SEC‐SWATH‐MS
Source: Mol Syst Biol. 2019 Jan 14;15(1):e8438. doi: 10.15252/msb.20188438 (PMC6346213; doi:10.15252/msb.20188438)
Supplement: Supplementary file 8 — Dataset EV7 [file MSB-15-e8438-s008.zip › feature_plots_string/Q9Y2X3.pdf]

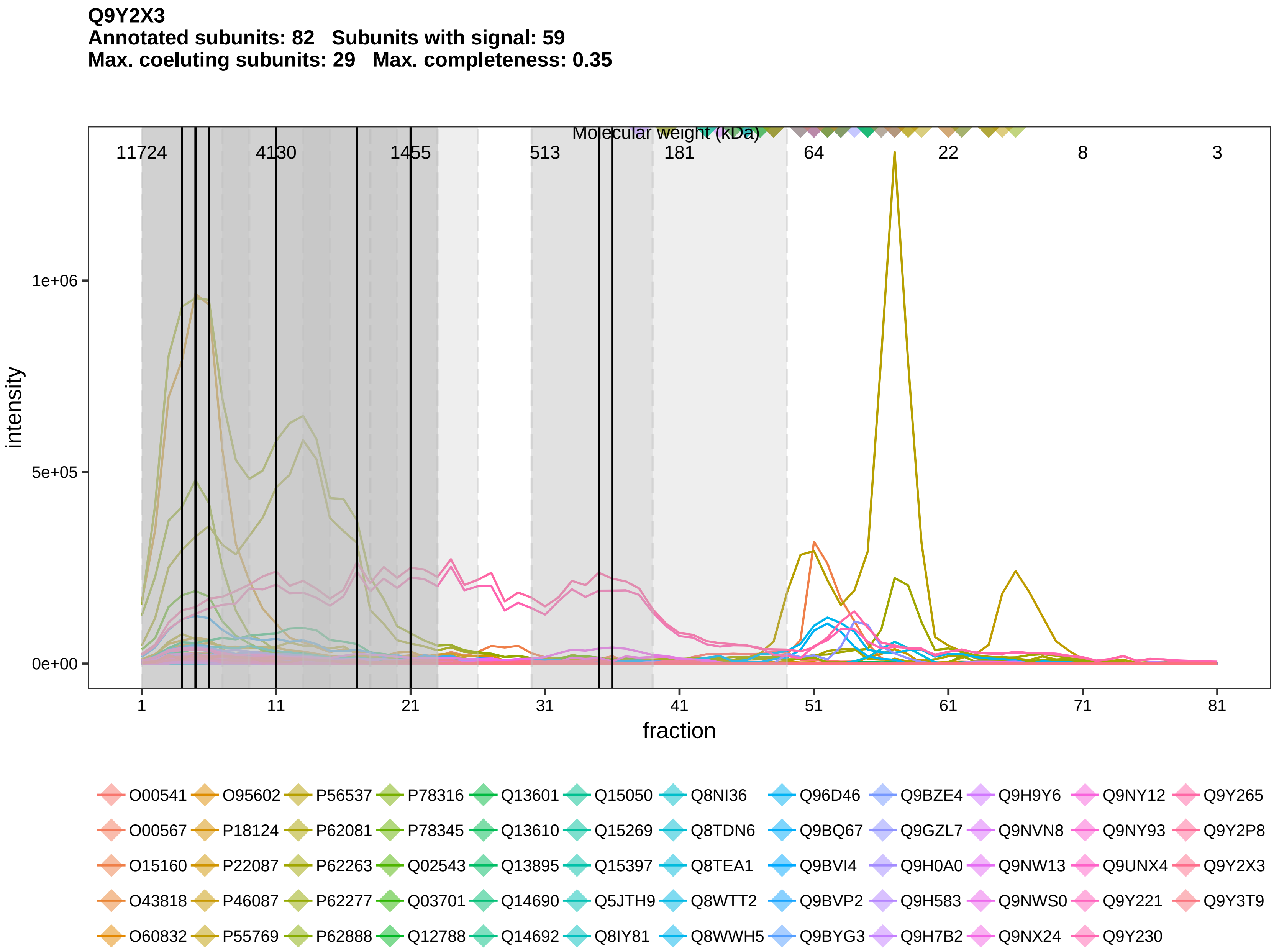

Q9Y2X3
Annotated subunits: 82 Subunits with signal: 59
Max. coeluting subunits: 29 Max. completeness: 0.35
Molecular weight (kDa)
11724
4130
1455
513
181
64
22
8
3
intensity
1e+06
5e+05
0e+00
fraction
1
11
21
31
41
51
61
71
81
O00541
O00567
O15160
O43818
O60832
O95602
P18124
P22087
P46087
P55769
P56537
P62081
P62263
P62277
P62888
P78316
P78345
Q02543
Q03701
Q12788
Q13601
Q13610
Q13895
Q14690
Q14692
Q15050
Q15269
Q15397
Q5JTH9
Q8IY81
Q8NI36
Q8TDN6
Q8TEA1
Q8WTT2
Q8WWH5
Q96D46
Q9BQ67
Q9BVI4
Q9BVP2
Q9BYG3
Q9BZE4
Q9GZL7
Q9H0A0
Q9H583
Q9H7B2
Q9H9Y6
Q9NVN8
Q9NW13
Q9NWS0
Q9NX24
Q9NY12
Q9NY93
Q9UNX4
Q9Y221
Q9Y230
Q9Y265
Q9Y2P8
Q9Y2X3
Q9Y3T9
